# Supplementary material for: Metabolomic and Transcriptomic Analyses Provide Insights into Metabolic Networks During Kiyomi Tangors Development and Ripening
Source: Plants (Basel). 2025 Sep 3;14(17):2751. doi: 10.3390/plants14172751 (PMC12430881; doi:10.3390/plants14172751)
Supplement: Supplementary file 1 [file plants-14-02751-s001.zip › Sup figures.pdf]

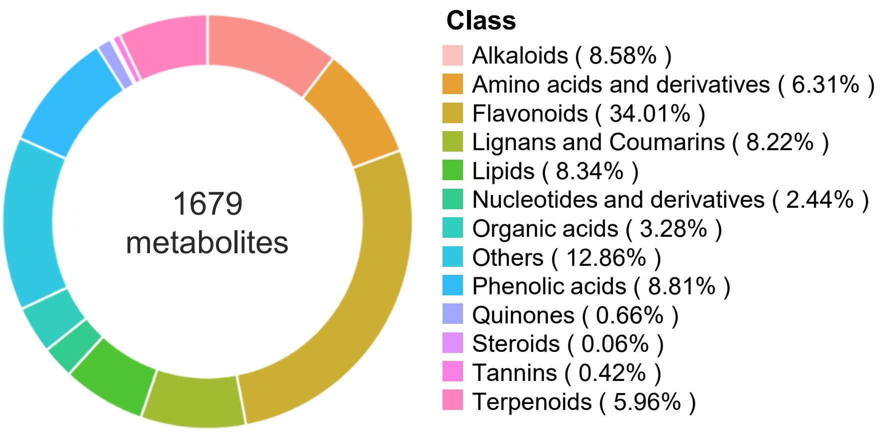

Figure S1. Classification of 1,679 identified metabolites based on their chemical classes.

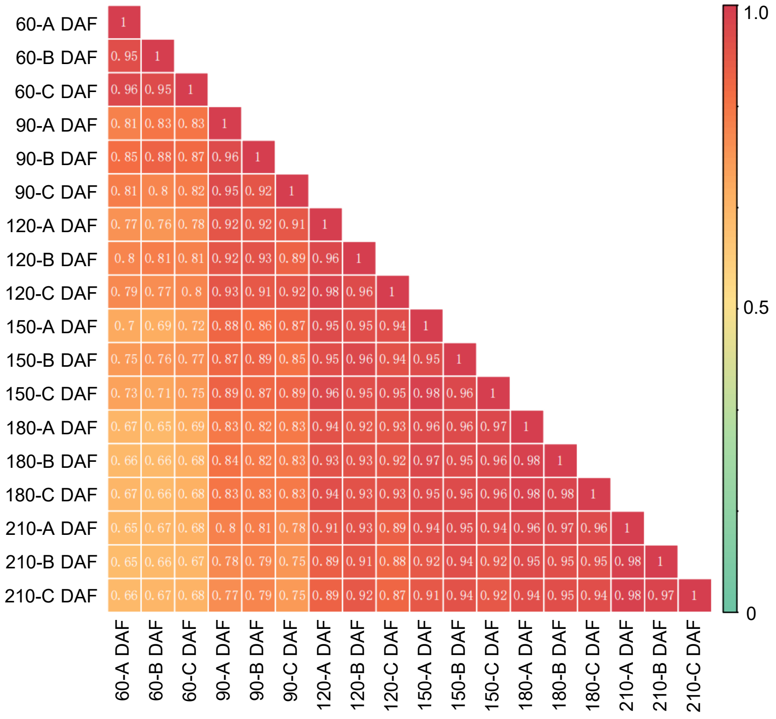

Figure S2. Correlation heatmap of 1,679 metabolites across six developmental stages.

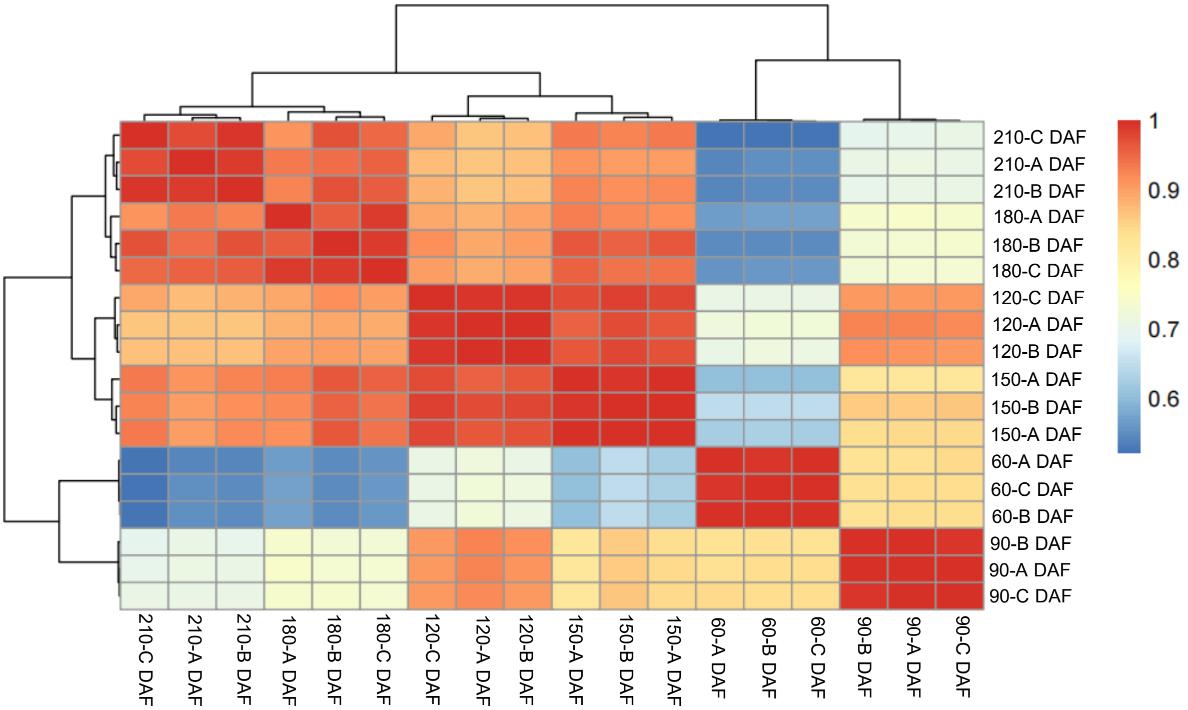

Figure S3. Pearson's correlation coefficients of RNA-seq datasets across six developmental stages of 'kiyomi' fruit.

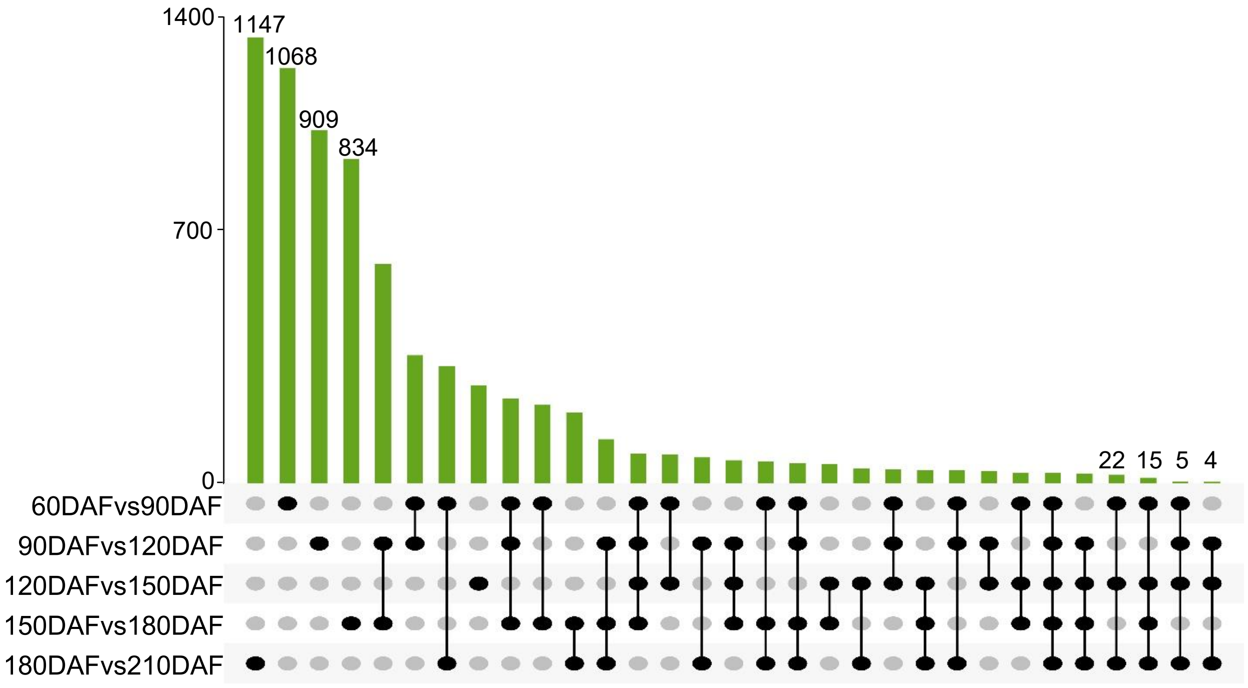

Figure S4. Upset plot of downregulated DEGs in pairwise comparisons between consecutive fruit developmental stages.
